# ‘Sustainable’ Rather Than ‘Subsistence’ Food Assistance Solutions to Food Insecurity: South Australian Recipients’ Perspectives on Traditional and Social Enterprise Models

**DOI:** 10.3390/ijerph15102086

**Published:** 2018-09-21

**Authors:** Sue Booth, Christina Pollard, John Coveney, Ian Goodwin-Smith

**Affiliations:** 1 College of Medicine and Public Health, Flinders University, Adelaide 5000, Australia; 2 Faculty of Health Sciences, School of Public Health, Curtin University, Perth 6102, Australia; C.Pollard@curtin.edu.au; 3 College of Nursing & Health Sciences, Flinders University, Adelaide 5000, Australia; john.coveney@flinders.edu.au; 4 College of Business, Government and Law, Flinders University, Adelaide 5000, Australia; ian.goodwinsmith@flinder.edu.au

**Keywords:** food assistance, food insecurity, food charity, food service, social enterprise models

## Abstract

South Australian (SA) food charity recipients’ perspectives were sought on existing services and ideas for improvement of food assistance models to address food insecurity. Seven focus groups were conducted between October and November 2017 with 54 adults. Thematically analysed data revealed five themes: (1) Emotional cost and consequences of seeking food relief; (2) Dissatisfaction with inaccessible services and inappropriate food; (3) Returning the favour—a desire for reciprocity; (4) Desiring help beyond food; and, (5) “It’s a social thing”, the desire for social interaction and connection. Findings revealed that some aspects of the SA food assistance services were disempowering for recipients. Recipients desired more empowering forms of food assistance that humanise their experience and shift the locus of control and place power back into their hands. Some traditional models, such as provision of supermarket vouchers, empower individuals by fostering autonomy and enabling food choice in socially acceptable ways. Improvement in the quality of existing food assistance models, should focus on recipient informed models which re-dress existing power relations. Services which are more strongly aligned with typical features of social enterprise models were generally favoured over traditional models. Services which are recipient-centred, strive to empower recipients and provide opportunities for active involvement, social connection and broader support were preferred.

## 1. Introduction

Despite comprehensive social welfare provisions in Australia, such as unemployment benefits and universal health care, increasing neoliberalism and economic pressures have resulted in insufficient and inadequate levels of income support for vulnerable groups [1]. The United Nations Committee on the Rights of the Child recommended that Australia improve its social services (for education, health, income support, disability services and employment to strengthen their responsiveness for those at risk) [1]. Liberal state welfare models increase reliance on markets, individual responsibility and charitable responses rather than the state acting to universally respect, protect and fulfil the needs of vulnerable citizens [2]. As a consequence of the liberal model of welfare capitalism [3], more Australians are experiencing poverty, leading to food inequality [4] and a reliance on food assistance. In countries without robust, adequate welfare safety nets, people rely on food relief provided by charitable organisations such as foodbanks, faith-based groups and non-government organisations [5,6].

South Australia has experienced an economic downturn due in part to key industries relocating, resulting in unemployment and population subgroups at increasing risk of food insecurity [7,8]. In 2015, approximately 75,000 South Australians (4.2% of the population) were classified as food insecure, with higher prevalence among: women (4.9% compared to 4.1% of men); the unemployed (12.3% compared to 2.2% of full-time employed); households with an income of less than AUD$20,000 (12.1% compared to 1.2% of income over AUD$80,000); and Aboriginal and Torres Strait Islander people (16% compared to 4.4% non-Indigenous) [9]. The demand for food relief has increased, with recipients described as socially isolated, homeless, unemployed, financially struggling and marginalised [10]. Complex client needs, intergenerational poverty, limited education and employment opportunities contribute to the demand [11]. There is also evidence of food insecurity and reliance on food charity among middle-income Australian families [12]. The growing number of ‘working poor’ may reflect the unaffordability of household utility costs [13].

Australian food relief is predominantly provided by charitable food services [6,14]. In 2015, the Federal government provided ~AUD$64 million to support the provision of emergency relief, which was estimated to provide food assistance for up to eight percent of the population [14]. State governments assist in managing the distribution of funds and may allocate additional grant funding for targeted programs, for example, school breakfast programs. Between 3000 and 4000 emergency relief services provided short-term, immediate food assistance to eligible recipients in 2015 [14]. Seventy percent of emergency relief agencies reported increasing demand for food assistance in 2016, up by eight percent since 2015 [15].

The effectiveness and appropriateness of the traditional charitable food assistance model, has been questioned by government and academics, in light of the increasing demand for food assistance and an emerging interest in social enterprise models [16]. The South Australian Government’s former Department of Communities and Social Inclusion (DCSI) (now Human Services) and SA Health commissioned research to explore recipients’ experience of charitable food services and their recommendations for service improvements.

Traditional food assistance models are delivered via partnerships between the non-profit sector and supermarket chains—often with some government funding—with the aim of redistributing food waste to those living below the poverty line [17]. Food services are diverse and include mobile soup vans, food parcels, supermarket vouchers, pantries, seated meal services, food hubs and food banks [6,14]. The food provided is usually donated by supermarkets to food banks, where it is collected by direct services or “rescued” and delivered by food rescue organisations and faith-based groups, or purchased directly from supermarkets. Food is usually provided to recipients free or at a minimal cost. There is limited information on the types of foods provided in Australia; however, internationally, the types of food provided by these types of services have been found likely to exacerbate recipients’ diet-related chronic disease conditions [6,18,19].

There are consistent reports from other jurisdictions of the recipients of traditional charitable food assistance being dissatisfied with the quality and quantity of food provided [20,21,22]. In addition to negative experiences due to limited food choice and poor food quality, recipients report feelings of shame, and describe the stigma and embarrassment associated with using food banks [23,24,25]. In Australia similar results are reported particularly concerning gratitude and shame: dissatisfaction with the variety, quality and types of food offered [26,27,28].

Social enterprise food assistance models, such as community or social supermarkets, social cafes, buying groups, and co-operatives, are uncommon in Australia, but are emerging as alternatives to the traditional charitable model [16]. Social enterprise broadly means ‘trading for a social purpose’, that is to say not for profit and for public benefit. However, there is little uniformity on what they are or do [29,30]. In other words, social enterprises are diverse, heterogeneous types of organisations using multiple activities to address the social needs of different client groups [31,32]. They vary in approach, but include: nonprofits’ income earning strategies; voluntary organisations contracted to deliver public services; democratically controlled organisations primarily aiming to benefit the community with limited profits for external investors; commercial businesses operating in public welfare fields or with a social conscience; and, locally driven community enterprises combatting a shared problem [29,30].

Market, government and voluntary sector failures have been identified as the reason for the lack of uptake of social enterprise models in Australia, even though social enterprise models have the potential to address all three failures [16]. When reviewing these models in Australia, Wills (2017) found that resistance to them may be a consequence of commercial stakeholders fearing devaluation of their product range, lack of government legislative support, and/or current legislation undermining practices that social entrepreneurs wish to take [16].

There is no research on Australian food assistance recipients’ perspectives on the likely benefits and limitations of social enterprise models to address food insecurity. Yet, the views of current food assistance recipients bring the lived experience perspective on receiving food assistance as well as helping to identify the elements of service delivery that are important to better meet needs. This study aimed to investigate recipients’ views on both of these approaches (traditional and social enterprise), compare food relief models and their perspectives on each model’s potential to meet the needs of food insecure people.

## 2. Materials and Methods 

This study used a qualitative focus group methodology. Ethical approval was granted by the Flinders University Social and Behavioural Research Ethics Committee (Project No. 7770).

### 2.1. Recruitment and Data Collection

The DCSI provided researchers with an email contact list of South Australian emergency food relief services which they fund. Purposive sampling was used to capture inner metropolitan, outer metropolitan and country areas, as well as a diversity of service types. This enabled researchers to capture multiple and different perspectives. An email was sent to the CEO or similar explaining the study. Of the twelve organisations invited, seven (2 inner city, 3 outer metropolitan and 2 country) were agreeable and provided the name and contact details of their service manager to assist with focus group recruitment. The researchers were in regular contact with service managers regarding the most convenient day, and time to run the focus group. During times of food relief operations, the service manager and researchers would randomly approach recipients, advise them of the study and invite them to participate. All service managers were invited to attend the focus groups which were run in conjunction with a scheduled food relief session. Three experienced researchers (SB, JC and I G-S) conducted the focus groups in pairs. Service staff approached food relief participants and invited them to participate using a standard verbal script outlining the time and location of the focus group. The focus groups were held on site in a private room approximately an hour later. Invitees were provided with a study information sheet and consent form and a verbal explanation was given and written consent was obtained before the focus group commenced.

Each group was digitally recorded, field notes were written up afterwards and a commercial service transcribed each group. Participants were given a AUD$30 supermarket gift card as a token of appreciation for their time and contribution.

### 2.2. Focus Group Guide

A semi-structured guide was developed by the research team to direct the discussion while allowing for diversions reflective of participants’ statements. Participants were asked to describe the type of charitable services they had used in the last year and the appropriateness and effectiveness of these services. A set of visual prompts were then used to assist participants to consider the pros and cons of traditional charitable and emerging social enterprise food relief service models. Finally, the group was asked to describe their ideal service for food relief provision.

### 2.3. Visual Prompts and Ranking of Preferences

**Figure 1:**
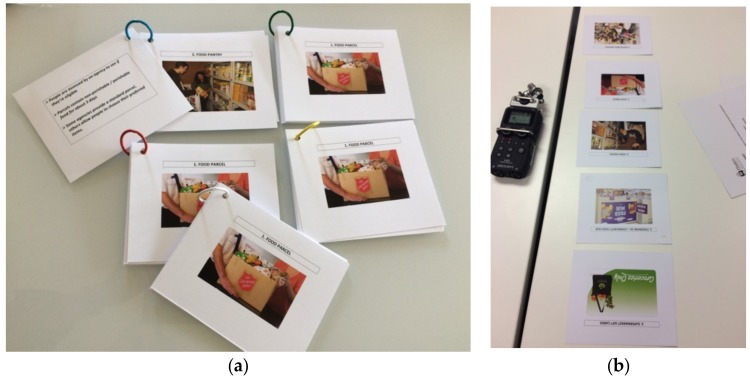
(**a**) The visual flash cards; (**b**) Visual flash cards in ranked order of preference during a focus group.

A set of eight pictorial flash-cards was developed by the researchers. Each card had a short description of the type of service on the back to assist participants in considering the pros and cons of a variety of different food assistance models. The cards were used to stimulate focus group discussion and for an assessment of overall preference for services (Figure 1). The cards were divided into 2 groups based on availability. The first five cards showed and described traditional charitable food relief options commonly available in South Australia: (i) Food parcels, (ii) Food pantries, (iii) Gift cards/vouchers, (iv) Seated meal services, and (v) and Foodbank Food hubs. The remaining three cards showed social enterprise models of food relief which were not available in South Australia, but examples existing interstate or internationally. These were (vi) Social café program, (vii) Food co-operatives, and (viii) Social supermarkets. Each Group was asked to place the cards in rank order, starting with the service type they would be least likely to use. In each focus group, discussion continued until consensus was reached on the preferential ranking of cards. The discussions during the group ranking exercise highlighted some of the potential positive and negative attributions of services.

### 2.4. Data Analysis

Focus group recordings and notes were transcribed and de-identified. CMP and SB read and re-read all the transcripts and a sub-sample were read by the remaining researchers. The data was then analysed using the qualitative software program QSR NVivo (version 11.4.3, QSR International, Doncaster, Victoria, Australia). Deductive codes were initially developed from the focus group schedule as well as from the researchers’ knowledge of the literature on experiences of charitable food service users. Inductive codes were developed from the focus group participant responses and discussed with the team to ensure rigour [33]. A thematic analysis was conducted by CMP with the codes and emergent themes cross-checked with the other members of the team until consensus was reached. SB double-coded three of the seven focus group transcript and any disagreements with the coding structure were discussed and the codes subsequently revised. Throughout the analysis, the data was further tested with the literature and subsequent focus groups in an iterative process. Data on the preferential ranking of service models across all focus group was tabulated. Verbal comments on the reasons for the ranking, pros, cons and recommendations to improve models were included in the thematic analysis.

## 3. Results

Fifty-four adults, 34 males and 20 females, who were recipients of food relief, participated in seven focus groups. Table 1 provides details of focus group location, service types from which participants were recruited and their gender.

Overall, participants had used nine different food relief models, often accessing multiple services to overcome service food restrictions on frequency and amount. Several vulnerabilities led participants to use charitable food services, these included: homelessness; precarious employment; low income due to insufficient welfare payments; relationship breakdown; gambling addiction; and rises in the cost of living. The long-term nature of the need for food relief was evident, for example the chronicity recorded in the field note of SB,

*“A woman on the far side of the table from me is of medium-thick build with shoulder-length strawberry blonde hair. She has broad facial features and makes intermittent eye contact. She tells the group she has been on the streets since she was 11 and she’s now about 41. She looks much older. She has diabetes and food allergies. After the focus group she says I look familiar to her. We work out that I interviewed her for my PhD on homeless youth and food insecurity in 2000. She is terribly excited by this and tells everyone in the vicinity… She shouts she can’t believe it and tells the people she is sitting with the story.”*

Field note extract, SB Focus Group 1.

Participants’ views on the pros, cons, and recommendations for improvement for the five traditional and three social enterprise food service models were varied and to some extent dependent on their current circumstances, as shown in Table 2.

Consensus on the preferential ranking of the five existing food service models varied across focus groups, depending on participants’ social circumstances. Preference also varied for the three new models presented; however, social supermarkets were ranked highest by half of the groups, see Table 3.

When participants ranked the traditional food assistance service models, the hub, supermarket voucher and pantry were the most preferred. Discussions of the pros and cons revealed that these models enabled choice and allowed recipients to behave as mainstream consumers, that is, to engage in socially acceptable methods of food procurement. These types of food service models were the most likely to create a sense of empowerment for those who used them. Participants’ recommendations to improve the traditional models generally focussed on universal eligibility or, if not possible, timely, dignified and respectful eligibility assessment processes. Traditional food assistance services also need to be re-engineered to provide the appropriate types and amounts of food to meet recipient’s physical needs, specifically for: family composition, nutrition requirements, duration of food insecurity; and availability of food preparation facilities. They believed that services should also be re-engineered to provide opportunities reduce social isolation and foster social connection over a meal, for example, to incorporate seated meal services including cafes.

When participants ranked the three social enterprise food assistance service models, the social supermarket was first, co-operatives second and social cafe third. Again, the preference was to engage in socially acceptable models of food procurement. When informed about these models, participants viewed them favourability, particularly the normalising of food procurement processes, and the opportunity for neighbourhood and community connection. Social supermarkets and co-operatives were viewed as offering a dignified eligibility process, as a member rather than a recipient, and the opportunity to access additional services to assist recipients out of food insecurity. The opportunity to visit a café was viewed as highly desirable but out of reach for most participants. The main barriers to the social café model were the short-term nature, agency eligibility assessment, and the fact that recipients could not bring along their family members.

**Figure 2:**
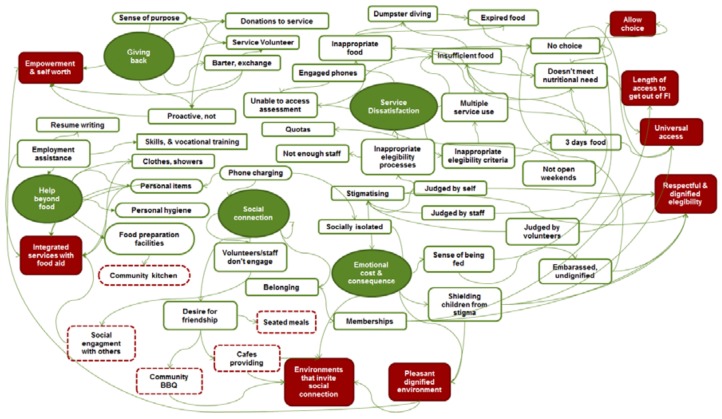
The thematic of food charity recipient perspectives on existing services and ideas for improvement—major themes (solid green), sub-themes (green outline), recipient recommendations (red outline), and final recommendations (solid red).

Five key themes emerged from all of the focus group and model ranking discussions (Figure 2). These were: (1) Emotional cost and consequences of seeking food relief; (2) Dissatisfaction with inaccessible services and inappropriate food; (3) Returning the favour—a desire for reciprocity; (4) Desiring help beyond food; and (5) “It’s a social thing”, the desire for social interaction and connection.

Each theme is described below.

### 3.1. Theme One: Emotional Cost and Consequences of Seeking Food Relief

Feelings of stigma, embarrassment, being judged or patronised as a result of many of the food service procedures or eligibility requirements. As one male said, *“I think the stigma should be the highlight I think and you* shouldn’t *be made to feel embarrassed because, you know, you’re sort of in need”.*

Negative comments regarding strict eligibility criteria for food relief were made in most groups and the notion of being referred by an agency to a particular service was seen as degrading and embarrassing,

*“Yeah, if you have more than two visits you’ve got to take all these documents and you asked all the questions ‘what do you do with the money, with the pension?’ They say ‘I get the same. How come I can do it and you can’t do it?”*

Male, Focus Group 1.

*“See, the problem with most of those is that, like you said, you have to go to an agency where they make you feel so degraded. They’re like ‘how much do you earn? What do you do with that money? Why don’t you have any money to buy food?’ and it is embarrassing whereas here you don’t have to explain yourself and the food co-op you don’t have to explain yourself, you go in, buy what you want or get what you want and you walk out the door. They’re not looking at you like ‘oh my God what.’”*

Female, Focus Group 2.

Operational inefficiencies had an emotional impact on recipients, contributing to their frustration and despair, for example, constantly engaged telephone lines that limited the opportunity for appointments to assess food assistance eligibility (Focus Group 6). One female participant showed the group her mobile telephone record of 39 calls logged trying to get an assessment appointment the morning of the interview. Delays in food eligibility assessments meant no food, and participants sometimes waited without food for up to three days for an appointment.

The impact of experiencing humiliation, judgment, embarrassment, or indignity during the food assistance process had some recipients stating they would not return to specific services, *“They make me feel this small. I never went there again.”* Male, Focus Group 1. 

Participants wanted services that were accepting and non-judgmental, “If I could go somewhere that did not make me feel degraded to ask for help, that’d be awesome.” Female, Focus Group 5.

Some types of food relief models were seen as less stigmatising, for example, supermarket gift cards,

*“Well, the card system is okay because there’s no sort of stigma, is not it? Some people feel sort of embarrassed or ashamed in entering places like these and if you go in a place like this and, you know, if a card is given to you, I mean you’re free to go and buy without no sort of stigma attached because nobody knows.”*

Male, Focus Group 2.

### 3.2. Theme Two: Inaccessible Services with Inappropriate Food

Food services were appreciated, but this was tempered by dissatisfaction due to problems related to food services, including: access, food types, the amount, and quality. Several participants mentioned that services were only available Monday to Friday and this made their life difficult, especially for those experiencing acute food shortages and needing emergency help.

*“Yeah I think just even general –like in general, not just food or whatever, for the homeless, for the shutting down on the weekend, you know, people have crises on weekends, people have crises—you know, it is a real business structure and does not conform to business hours.”*

Female, Focus Group 2.

Supermarket vouchers and gift cards usually had a AUD$20 value. The amount was considered meagre and, although possibly suitable to pay for an individual’s food for one day, the amount was viewed as insufficient for a family,

*“They [cards] are very useful but realistically what can you get out of them, $20? I have a family of eight.”*

Female, Focus Group 4.

Some participants said that the food offered was generally poor or may have exacerbated their existing health conditions, for example, diabetes, eating disorders, irritable bowel, allergies or mental health issues.

*“I’m allergic to tomatoes and things like that so literally for myself, like trying to find—something like macaroni and cheese for me would be all right but I’m not supposed to eat too much pasta because of my diabetes so I’d have to actually find a way of being able to split that into two because two nights of—that whole meal would put me in hospital because of how much gluten’s in it so I’m probably queen of the fuss but I’d just still rather be healthy by what I’m eating as well.”*

Female, Focus Group 1.

*“Pies are bad for diabetics because of the pastry. Pastry is really bad.”*

Female, Focus Group 1.

Ready-made emergency food parcels also caused concern for people with existing health conditions because participants said they may contain food they were unable to eat. Negotiating to change the parcel contents or asking for a supermarket voucher instead caused some participant to experience guilt,

*“… if I was to say ‘look, I’ve got food issues and the food parcel that you’ve just given me, I really can’t eat anything out of that’ I would feel really—I feel guilty getting a [supermarket voucher/ gift] card.”*

Female, Focus Group 2.

Supermarket vouchers and food pantries were preferred over ready-made food parcels because they afforded the opportunity for individuals to choose their own food items, with the statements below being typical:

*“if you’ve got the card (supermarket gift card), you feel free to pick up what you want …”*

Male, Focus Group 2.

I like the … *“Food pantry because then you get to choose it.”*

Female, Focus Group 1.

Some service aspects and management strategies seemed to work better for recipients,

*“… we paid $2. You get a little card. For every dollar you spend it gets accumulated up and at a Christmas time that amount would come off a Christmas hamper that they’d do, which I thought was absolutely fantastic. Go in there if you’ve made anything. If you’ve grown veggies or fruit you could take it in there and they give back to you. Like you give them that, you can get a couple of meals.”*

Female, Focus Group 4.

To enable individuals to better manage their food insecurity, rather than seek help from food relief agencies, one Focus Group suggested a person’s entire welfare payment should be directly deposited into supermarkets. They said this would avoid the money for food not being available if individually managed,

*“You give it to—whatever shopping centre you use, you give it to them, the whole cheque, and so, okay, you can spend this much every week or every fortnight. You can’t spend the whole lot in one hit. That will allow people to go there when they need something for tea or breakfast or whatever. They can grab the specific items and then go home ‘oh well, I can make a meal and everything now’ and then tomorrow comes ‘well, I need this. I need onions and I need carrots and stuff’. Go in and buy them because if you gave someone a cheque they’d just go in and blow the whole lot.”*

Male, Focus Group 1.

### 3.3. Theme Three: Returning the Favour—Reciprocity

Some participants engaged with services as both volunteers and recipients of food relief. Others desired to volunteer at services; however, the degree to which both men and women wanted to reciprocate at a ‘pop up’ service was sometimes unable to be accommodated. One comment indicated that children had also offered to volunteer at this same service. The quotes below are illustrative,

*“We have on average maybe seven requests to volunteer every week. Well we don’t [take more volunteers on]—I mean we’re not ungrateful but we don’t need them …”*

Female, Focus Group 6.

*“I volunteer and then sort of just pick some bits and pieces during my shift to take home to help out the family. I also am on a single parent pension so coming here for food relief by the volunteering …”*

Female, Focus Group 7.

For some, the volunteering at a food relief service was driven by a desire to reciprocate for the assistance they had received.

*“(I was) one of the people that lined up every week and then I started volunteering and I actually like returned the favour, giving back to the community.”*

Female, Focus, Group 5.

Duties included staffing the café, preparing and/or re-packaging food items for distribution, or chatting to people lining up. Volunteers empathised with the circumstances and feelings of those seeking food relief variously commenting,

*“We’re not judging them”, and “we don’t hold ourselves higher than what they are. We’re one of them.”*

Female, Focus group 5.

Potential volunteer duties at food relief services were variable with garden maintenance on the premises suggested. Others suggested donating fresh home grown produce to food relief services as an acknowledgement of previous help or using produce to barter for pre-prepared meals or other food items,

*“… people could be working on an idea of providing food for—and people could contribute to that facility, if you know what I mean, and providing food for people on a regular basis, so people would be going there for meals and working in the garden and socialising …”*

Male, Focus Group 2.

*“‘oh the lemon tree is nearly ready for picking’ and then they go ‘oh we’ll bring lemons’. You know, big boxes like this that they’ve walked down the street with in wheelbarrow … things or oranges, mandarins. If people have got trees they bring in the stuff to share back, like to say thank you to us.”*

Female, Focus Group 5.

*“Go in there if you’ve made anything. If you’ve grown veggies or fruit you could take it in there and they give back to you. Like you give them that, you can get a couple of meals.”*

Female, Focus Group 4.

### 3.4. Theme Four: Help Beyond Food

Many participants described a need for help with other parts of their lives. For example, there was a need for items like nappies and toiletries, homeless people needed access to showers, laundry facilities and phone charging, domiciled people needed white goods (fridges, washing machines). Phone charging was important to keep people connected and the removal of free power points in the city mall was noted. Alternative charging stations were used including at McDonald’s restaurants, suburban train carriages, some street locations, and libraries. Existing shower services were sometimes described as “hopeless” and the lack of showers in places like parklands were noted, prompting suggestions for alternative showers,

*“Most people will pay … $20 and go to the bus terminal, give $20 over because it is a $20 deposit for the key and get the key and you can have a shower as long as you like. Then you go hand your key back and get your $20 back. That is how a lot of people get their showers …. And sometimes they let you put your phone [in] and go in the shower.”*

Male, Focus Group 1.

“Can I just say something about the showers? I was talking to Orange Sky* not long ago because Orange Sky showers are going to be hopefully starting in January.”

Male, Focus Group 1 (Orange Sky is free of charge mobile laundry service).

Some participants wanted financial assistance and management, others were interested in support to increase the likelihood of employment such as resume development or assistance from a social worker.

*“… you go to Centrelink and they go ‘oh, it is nice if you start looking for work’—like before I had the babies—and it is like well, how do you put a resume together?”*

Female, Focus Group 4.

*“… there needs to be like a social worker or someone that can be there to listen …”*

Female, Focus Group 4.

There were positive comments on the social supermarket model, centred around learning and training opportunities, which offered pathways out of poverty and a reliance on food relief.

*“I really like the fact that with that one you’re actually doing something as well. I just think that anything where people can learn to become better …. I would see it as a building and a stepping stone even more so because you get training …. They’re the sort of things and for me that gives a person hope. That builds hope that I can get out of this position and get a bit better in life. You know, that I love.”*

Female, Focus Group 7.

These suggestions, along with giving back, indicate a desire to gain skills and resources to seek financial independence or employment and get out of poverty.

### 3.5. Theme Five: “It’s a Social Thing”—Desire for Social Interaction and Connection

In seeking food assistance, participants simultaneously sought meaningful social interaction and connection in a friendly atmosphere. They described how they wanted opportunities to engage with other regulars and volunteers, laugh, converse over coffee, or a meal or engage in fun activities.

*“I’d rather the person that is handing the meal out, when you’ve finished handing the meals out sit down and talk to us, spend time with us. Don’t just hand the meal out and go ‘zoom’ and take off and go somewhere else.”*

Male, Focus Group 4.

*“I’d rather go to [Service] because they sit down and talk and laugh and have fun and everything like that.”*

Male, Focus Group 5.

Participants who had used food relief and who now volunteered their time registered the importance of providing social support for recipients,

*“We get a lot of people that don’t come here for the food; they come here because they know they can have a chat. Sometimes we’re the only people in the whole entire week that they’ve spoken to …”*

Female, Focus group 5.

An outcome of social connection at food relief services included the development of friendships, and in one case marriage and cohabitation,

*“Those people that are getting married, be it that they’re elderly but they were facing nursing homes because they had no-one to look after them. They were living on their own and they met here. They’ve now moved in together. They don’t have to move into nursing homes.”*

Female, Focus Group 8.

## 4. Discussion

The aim of this study was to investigate food assistance recipients’ views on both existing services in South Australia and on examples of social enterprise models. We also sought to understand how food assistance might be improved more broadly to better meet the needs of food insecure people. Five themes emerged from the discussions: (i) considerable emotional costs and consequences in receiving food assistance, (ii) dissatisfaction with inaccessible services and inappropriate food, (iii) desire to reciprocate for food assistance by volunteering at services, (iv) the need for help goes beyond food, and (v) a strong desire for social connection.

Participants desired food assistance models that afforded some of the features characteristic of social enterprise models, particularly the opportunity to exercise food choice, meet their desire for social connection and commensality, and provide access to other services such as training or skills development. Social supermarkets offer an innovative model of food assistance which could address some of these points, but are unavailable in Australia [34]. Internationally, there are a variety of novel food assistance practices that have the potential to transform incrementally and interact with other food systems to deliver pathways out of food poverty [35]. Our work suggests that recipients support the re-making of traditional food relief models as a way to support individual empowerment and pathways out of food insecurity.

The findings highlighted the power imbalances inherent in the provision of food assistance and suggests that they are deeply embedded at an operational level in existing South Australian services. The intrinsic design and delivery of charitable food assistance can be either disempowering or empowering. Forms of assistance which are empowering help vulnerable people climb out of their neediness and offers real pathways out of food insecurity. In contrast, disempowering assistance traps clients in a continuous, chronic food assistance cycle.

This study found evidence of disempowerment within traditional South Australian food charity models such as stigma or embarrassment, having to prove their eligibility, need or worthiness for assistance. Empowering options aligned more strongly with social enterprise models.

### 4.1. Disempowering Food Assistance

Although recipients were grateful for food assistance, there were several aspects of the system that were experienced as disempowering, which contributed to the emotional costs and consequences in receiving food relief including loss of power, similar to those described by van der Horst et al. (2014) [36]. Aspects of traditional food assistance models inadvertently impact the emotional wellbeing of recipients by fostering negative feelings such as judgment, embarrassment and stigma. The emotional consequences of having to ask for food assistance in the first place speaks to an admission of failure that one cannot provide food for oneself. This can be so overwhelming for some people that they would rather avoid seeking food assistance [36]. These findings are consistent with evidence from other wealthy industrial countries for recipients of food bank users and other types of food charities [25].

The power imbalance is also evidenced by the dissatisfaction with inaccessible and inappropriate food services, particularly recipient’s inability to enforce their right to food, their freedom to choose food the food they want in socially acceptable ways, or eat in dignified settings. Riches (2018) asserts that it is “the universal right of vulnerable individuals and families to be able to feed themselves with choice and human dignity” (p. 3) [37]. Recipients were frustrated with the lack of choice in the current system and desired the dignity of being able to choose their own food, recommending models that were considered empowering and less stigmatising, such as supermarket gift cards. These finding were consistent with those of recipients in Perth, Western Australia [27].

### 4.2. Empowering Food Assistance

Study participants expressed a desire to receive flexible, recipient-oriented services that were empowering, encouraged independence and autonomy. They had a strong desire for giving back—that is, wanting to ‘return the favour’—for example, by volunteering at services when their circumstances allowed them to. Applying Mauss’s 1925 framework of gift exchange, food [charity] is this context is essentially a gift which cannot be reciprocated and may render the recipient powerless [38]. Inherent in Mauss’s theory of gift giving is the obligation to reciprocate. This may explain the strong desire of participants to ‘return the favour’, namely, to regain a modicum of situational power by donating fruit or volunteer labour.

Recipients described the need for ‘Help beyond food’. Their desire for empowerment went beyond food. Participants spoke positively of food assistance models that extended to the social purpose of tackling food insecurity and offered a viable pathway out of chronic reliance on food assistance. The current findings align with the UK’s All-Party Parliamentary Group (AAPG) recommendations on the Hunger and Food Poverty inquiry into foodbanks in the UK [39]. The AAPG called for models to end food poverty which were ‘sustainable’ rather than offering ‘subsistence’ and recommended a ‘food bank plus model’, as described by Paget et al. (2015) [40]. The nature of the ‘food plus model’ included multiple services, all of which should be considered when reviewing funding to food assistance services in South Australia.

In Australia, charitable food assistance services rely on foodbanks and food rescue organisations to redistribute retail food waste. The participants in this current study, although grateful, were dissatisfied with the food provided by services, describing issues with the appropriateness and quality of food and the reliance on charity, and ultimately their inability to attain a varied and healthy diet in an autonomous way. The conversations rang true to the sentiment of ‘Left over food for left over people’ previously described by Dowler [41]. Participants wanted to ‘fit in’ and to shop at supermarkets and eat at cafes like ‘normal people’, and they did not want their children to know they were struggling. The findings suggest the retail sector reconsider their moral and social obligation in light of the right to food for the most vulnerable citizens residing in countries where they operate. For example, as part of their retail practice they could directly provide dignified access to appropriate food by assisting people during times of economic hardship to access their goods in socially acceptable ways.

The current study findings also highlight the acceptability of some of the aspects of social enterprise models to address food insecurity among recipients. A well-developed example of a social enterprise model to food insecurity are social supermarkets (SSMs). SSMs are a retail formula where the outlet receives free surplus food and consumer goods from partner companies and sells them at symbolic prices to people who are at risk of, or living in poverty (Holweg and Lienbaucher 2010) [34]. They may also operate as retail training grounds to assist people who are long term unemployed or disabled re-integrate into society. In doing so, SSMs provide opportunities for work and immediate positive fulfilment and feedback; they provide a wage rather than government handouts and subsidies, and they build individual confidence and resilience [42]. SSMs are widespread in continental Europe, with more than 1000 in operation in 2013 [43], but few, if any, examples exist in Australia. Despite being widespread, however, there is no available literature evaluating the effectiveness of social supermarkets.

Successful programs for food assistance and other support pathways are likely to be ones that are co-produced with recipients [39] and the current findings highlight the value in obtaining recipient reviews on current and future service options. Co-production has become synonymous with innovative approaches to service delivery and been defined as “A meeting of minds coming together to find a shared solution. It involves people who use services being consulted, included and working together from the start to the end of any project that affects them” (p.7) [44].

### 4.3. The Desire for Social Interaction and Connection

Participants desired meaningful social interaction and connection, recognition and acknowledgement, and friendship networks. The sense of isolation and loneliness experienced by homeless people or those living in poverty is well documented and may constitute a risk to survival [45]. Loneliness is adversely associated with physical and mental health and lifestyle factors [46]. The experience of social pain, defined as the unpleasantness that is associated with actual or potential damage to one’s sense of social connection or social value (owing to social rejection, exclusion, negative social evaluation or loss) may involve an overlap of the neural circuitry underpinning physical pain (defined as the unpleasant experience that is associated with actual or potential tissue damage) [47].

High levels of concern about the consequences of loneliness experienced by all ages has prompted calls for it to be considered a public health issue [48]. The strong preference for seated shared meal services, commensality and connection with others in the current study suggest that social enterprise models integrated with cafes and restaurant dining are an option [49,50].

The study has several strengths and limitations. A strength of this study is that the 54 participants were recipients of food assistance from different geographic locations (metropolitan, regional and country areas) in South Australia. They provided a real-life perspective on the issues and potential solutions. The presentation of three novel social enterprise options to provide food assistance enabled participants to think beyond the current system; however, as there are few social enterprise options in Australia [16], they did not have an experience of using these types of services. Only three social enterprise models were presented, with very little description (one image and three to four descriptive sentences), further research is needed to identify and pilot the effectiveness of social enterprise models for food assistance in Australia.

A limitation of the current research is that it was not designed to explore options to address food insecurity other than food assistance. There are numerous social and economic policy actions that should be explored as to their effectiveness in addressing food insecurity, for example, increasing the minimum welfare payments, employment schemes or other economic options that are under the auspice of government. There was a noticeable absence of government policy and/or accountability in the food assistance system in South Australia, and indeed in Australia. Further research is needed to describe options for an integrated food assistance system that includes government, commercial sector and voluntary organisations.

The findings suggest that the retail sector may have an important role to play in addressing food insecurity, outside the current food waste redistribution paradigm. Recipient dissatisfaction with the food currently available suggests that food acquisition and distribution models need to be critically analysed for their ability to address food insecurity. Exploration of effective Corporate Social Responsibility commitments to address food insecurity that are not reliant on redistributing waste food is warranted.

## 5. Conclusions

Food systems, including charitable food systems, need to work for everyone, especially those who are vulnerable. This study has revealed aspects of the existing South Australian food assistance system that can be disempowering to recipients. Disempowering forms of food assistance can trap recipients in a cycle of food charity. Participants desired empowering forms of assistance that humanise the charitable food system, shift the locus of control and place power back into the hands of users. Improvement in the quality of existing food relief models, should focus on recipient-informed models (co-production) which re-dress existing power differentials. Services which are more strongly recipient-centred, strive to empower clients and provide opportunities for active involvement, social connection and broader support are needed.

## Figures and Tables

**Table 1:** Location of focus group services, brief description, date and number of participants.

Group	Location	Service Description	Participants (*n*) Gender Split
1	Inner city	Seated breakfast program and emergency food relief appointments (Voucher)	(10) 7 men, 3 women
2	Inner city	Emergency food relief appointments (Food pantry access), free bread service	(7) 6 men, 1 woman
3	Country	Emergency food relief appointments (Food hub)	(7) 5 men, 2 women
4	Country	Emergency food relief appointments (Food parcels)	(4) 2 men, 2 women
5	Outer metropolitan	Volunteer run food hub—free food plus access to some items at reduced prices	(9) 2 men, 7 women
6	Outer metropolitan	Emergency food relief appointments (Food pantry access)	(7) 5 men, 2 women
7	Outer metropolitan	Food pantry, free bread, fruit and vegetables	(10) 3 men and 7 women

**Table 2:** Participants’ perspectives on the pros and cons of food service models and their recommendations to improve them.

Model	Pros	Cons	Recommendation
**Food Parcel**	Commonly available Grateful for parcels when have nothing	‘Harsh’ eligibility criteria Inappropriate amount of food for family, types of food for special diets Inadequate nutritious foods No choice Short term (1–3 day) solution Food expires if you get more Incomplete meals—no meat Homeless people cannot carry	Respectful and dignifying eligibility processes Appropriate amounts and type of food to suit nutrition needs (e.g., meat, recipes, full meals, nutritious foods, length of time to cover) Ability to choose items
**Food Pantry**	Allows choice Other items available (toiletries, washing powder etc.) Fresh produce Suitable if have access to cooking facilities	Limits to number of items Close to expired food Limited types of foods, e.g., meat Can only use twice a year Must prove need Difficulty securing an assessment appointment Insufficient daily appointments Have to waiting for appointment despite immediate need Not suitable if no cooking facilities	Respectful and dignifying eligibility processes Reduce appointments waiting time, e.g., free calls or 1800 number Appropriate amounts and type of food to suit nutritional needs (meat, recipes, full meals, nutritious foods) Align food quantity with need Increase access during holidays and weekends
**Supermarket gift card**	Allows choice Can buy other essential items Easy to carry Dignifying and ‘normal’ way to acquire food	‘Harsh’ eligibility criteria Amount ($20) is inadequate Only allowed to spend at major supermarket chains where food is expensive	Increase supermarket voucher card value Relax eligibility criteria Cash for purchases from alternative food businesses
**Seated meal services**	Best for people without dwelling, social isolated or cooking facilities Able to combine with other services (e.g., shower, phone charging) Social engagement with volunteers	Families with young children too noisy Sometimes unpleasant environment/people Do not want children to experience the stigma Cost to recipient Can miss out on food because there is not enough and waiting time is too long No-one sits down to and talks to you Agency referral needed	Combine with other services Maintain pleasant, quiet, dignified atmosphere Tailor food service to client needs Universal eligibility Socially connect with recipients
**Foodbank Food Hubs**	Membership-based Reward/incentive program Discounts towards end of year, pre-saving for Christmas hampers Free bread, fruit and vegetables	Agency-issued vouchers require assessment appointments. Viewed as judgemental, embarrassing and undignified Food that is unsaleable or approaching its use by date or expired.	Universal eligibility or respectful and dignifying eligibility processes Membership includes rewards scheme for every dollar spent Food is purchased using own money Blended model—free food and some discounted for purchase Increase access during holidays and weekends
**Co-operative**	Dignifying Dietitian assessed low-cost food packs with recipes for preparing at home Offer toiletries, toys etc. Best with other services including seated meals	Membership fee Having to pay for food if no income	Include other services, e.g., seated meals or cafes Make it more accessible to people e.g., transport Increase access—Open during school holidays, weekends and major holidays
**Social cafe**	Allows access to mainstream café—normalising experience Helps isolated individuals An outing for a special occasion	Agency eligibility and assessment Meal subsidy is time limited Does not allow for family members and children Dependent on participating café in local area	Universal eligibility or respectful and dignifying eligibility processes Incorporate access for children and family members Free community barbecues to reduce social isolation and provide a treat/family outing
**Social supermarket**	Opportunity for capacity building and volunteerism Associated café providing cheap meals Membership and discounted food Supermarket style format, can exercise individual food choice Other services can be accessed via the social supermarket—the idea of linked service valued One-stop shop Opportunity for socialisation, community connection	Stocked with food that may be expired or close to use by date. Purchased food may have a shorter life span?	Increased access—Open during school holidays, weekends and major holidays

**Table 3:** Focus group consensus ranking scores for participants’ preference for five traditional models and three social enterprise food service models.

Ranking *	Traditional Models					Social Enterprise Models		
Focus group	Hub	Voucher	Pantry	Seated Meal	Parcel	Social Supermarket	Co-op	Social Café
1	5	2	3	1	4	2	3	1
2	5	4	3	2	1	2	1	3
3	1	2	3	5	4	1	3	2
4	2	1	3	5	4	1	3	2
5	1	4	3	2	5	1	2	3
6	2	1	3	5	5	2	1	3
7	1	3	2	5	4	1	2	3
Total	17	17	20	25	27	10	15	17

* Traditional models ranked from 1 most preferred to 5 least preferred (total possible 35) and social enterprise models from 1 most preferred to 3 the least preferred (total possible = 21).

## References

[1] United Nations Committee on the Rights of the Child Sixtieth Session 29 May–15 June 2012. http://docstore.ohchr.org/SelfServices/FilesHandler.ashx?enc=6QkG1d%2fPPRiCAqhKb7yhsk5X2w65LgiRF%2fS3dwPS4NXPtJlvMuCI3J9Hn06KCDkN8AgEcc%2bNlwRMULqb84PSl9FicZROAZolAudnAZ3CxmRZ%2fzxW2Yn8qOrVcMCd9xFL.

[2] Claeys P. (2015). The right to food: Many developments, more challenges. Can. Food Stud..

[3] Esping-Andersen C. (1990). The Three Worlds of Welfare Capitalism.

[4] Pollard C., Begley A., Landrigan T. (2016). The rise of food inequality in Australia. Food Poverty and Insecurity: International Food Inequalities.

[5] McKee M., Reeves A., Clair A., Stuckler D. (2017). Living on the edge: Precariousness and why it matters for health. Arch. Public Health.

[6] Pollard C.M., Mackintosh B., Campbell C., Kerr D., Begley A., Jancey J., Caraher M., Berg J., Booth S. (2018). Charitable food systems’ capacity to address food insecurity: An Australian capital city audit. Int. J. Environ. Res. Public Health.

[7] Jericho G. (2018). Why South Australians Are Older, Poorer and on Their Way Interstate. The Guardian Newspaper.

[8] BankSA, Manufacturing South Australia’s Future (2017). Trends—November 2017—A Bulletin on Economic Development in South Australia. https://www.banksa.com.au/content/dam/bsa/downloads/bsa-media-trends-nov-2017.pdf.

[9] Anglicare (2017). Improving Individual and Household Outcomes in South Australia—Discussion Paper.

[10] Wingrove K., Barbour L., Palermo C. (2017). Exploring nutrition capacity in Australia’s charitable food sector. Nutr. Diet..

[11] McKay F., McKenzie H. (2017). Food aid provision in metropolitan Melbourne: A mixed methods study. J. Hunger. Environ. Nutr..

[12] Kleve S., Davidson Z., Gearon E., Booth S., Palermo C. (2017). Are low to middle income households experiencing food insecurity in Victoria, Australia? An examination of the Victorian Population Health Survey 2006–2009. Aust. J. Prim. Health.

[13] Ramsay R., Giskes K., Turrell G., Gallegos D. (2012). Food insecurity amongst adults residing in disadvantaged urban areas: Potential health and dietary consequences. Public Health Nutr..

[14] Lindberg R. (2015). Still serving hot soup? Two hundred years of a charitable food sector in Australia: A narrative review. Aust. N. Z. J. Public Health.

[15] Foodbank W.A. Foodbank WA Annual Report 2015. https://www.foodbankwa.org.au/wp-content/blogs.dir/5/files/2015/10/Annual-Report-Final-2015_web.pdf.

[16] Wills B. (2017). Eating at the limits: Barriers to the emergence of social enterprise initiatives in the Australian emergency food relief sector. Food Policy.

[17] Richards C., Kjaernes U., Vik J. (2016). Food security in welfare capitalism: Comparing social entitlements to food in Australia and Norway. J. Rural Stud..

[18] Seligman H., Lyles C., Marshall M., Prendergast K., Smith M., Headings A., Bradshaw G., Rosenmoss S., Waxman E. (2015). A pilot food bank intervention featuring diabetes appropriate food improved glycemic control among clients in three states. Health Aff..

[19] Miewald C., Ibanez-Carrasco F., Turner S. (2010). Negotiating the local food environment: The lived experience of food access for low-income people living with HIV/AIDS. J. Hunger. Environ. Nutr..

[20] Hamelin A., Beaudry M., Habicht J. (2002). Characterization of household food insecurity in Quebec: Food and feelings. Soc. Sci. Med..

[21] McNeill K. (2011). Talking with Their Mouths Half Full: Food Insecurity in the Hamilton Community. Ph.D. Thesis.

[22] Loopstra R., Tarasuk V. (2012). The relationship between food banks and household food insecurity among low-income Toronto families. Can. J. Public Health.

[23] Garthwaite K. (2016). Stigma, shame and ‘people like us’: An ethnographic study of foodbank use in the UK. J. Poverty Soc. Justice.

[24] Purdam K., Garrett E.A., Esmail A. (2016). Hungry? Food insecurity, social stigma and embarrassment in the UK. Sociology.

[25] Middleton G., Mehta K., McNaughton D., Booth S. (2018). The experiences and perceptions of foodbank amongst users in high income countries: An international scoping review. Appetite.

[26] Booth S. (2003). Eating Rough—Food Insecurity Amongst Homeless Young People in Adelaide. Ph.D. Thesis.

[27] Booth S., Begley A., Mackintosh B., Kerr D.A., Jancey J., Caraher M., Whelan J., Pollard C.M. (2018). Gratitude, resignation and the desire for dignity: Lived experience of food charity recipients and their recommendations for improvement, Perth, Western Australia. Public Health Nutr..

[28] Middleton G. (2015). Evaluation of a Community Food Banking Model in South Australia. Honours Thesis.

[29] Teasdale S. (2011). What’s in a name? Making sense of social enterprise discourse. Public Policy Adm..

[30] Teasdale S. (2010). How can social enterprise address disadvantage? Evidence from an inner city community. J. Non-Profit Public Sect. Mark..

[31] Shaw E., Carter S. (2007). Social entrepreneurship: Theoretical antecedents and empiricle analysis of entrepreneurial processes and outcomes. J. Small Bus. Enterp. Dev..

[32] Sharir M., Lerner M., Yitshaki R., Robinson J., Mair J., Hockerts K. (2009). Long term survivability of social ventures: Qualitative analysis of external and internal explainations. International Perspectives of Social Entrepreneurship.

[33] Henninck M., Hutter I., Bailey A. (2011). Qualitative Reseach Methods.

[34] Holweg C., Lienbacher E., Zinn W. (2010). Social supermarkets-a new challenge in supply chain management and sustainability. Supply Chain Forum.

[35] Hebinck A., Galli F., Arcuri S., Carroll B., O’connor D., Oostindie H. (2018). Capturing change in european food assistance practices: A transformative social innovation perspective. Local Environ..

[36] van der Horst H., Pascucci S., Bol W. (2014). The “dark side” of food banks? Exploring emotional responses of food bank receivers in the netherlands. Br. Food J..

[37] Riches G. (2018). Food Bank Nations. Poverty, Corporate Charity and the Right to Food.

[38] Mauss M.T. (1990). The Gift: The Form and Reason for Exchange in Archaic Societies.

[39] All-Party Parliamentary Group on Hunger and Food Poverty (2014). Feeding Britain: A Strategy for Zero Hunger in England, Wales, Scotland and Northern Ireland, the Report of the All-Party Parliamentary Inquiry into Hunger in the United Kingdom.

[40] Paget A. (2015). Community Supermarkets Could Offer a Sustainable Solution to Food Poverty.

[41] Caraher M., Furey S. (2018). The Economics of Emergency Food Aid Provision: A Financial, Social and Cultural Perspective.

[42] Schneider F. (2013). The evolution of food donation with respect to waste prevention. Waste Manag..

[43] Cocozza P. ‘If I Shop Here I’ve Got Money for Gas’: Inside the UK’s First Social Supermarket. http://www.theguardian.com/society/2013/dec/09/inside-britains-first-social-supermarket-goldthorpe-yorkshire.

[44] Council of the Ageing (2014). The Voice of Consumers in Home Care: A Practical Guide. https://www.cota.org.au/publication/the-voice-consumers-in-home-care-guide/.

[45] Pantell M., Rehkopf D., Jutte D., Syme S.L., Balmes J., Adler N. (2013). Social isolation: A predictor of mortality comparable to traditional clinical risk factors. Am. J. Public Health.

[46] Richards A., Rohrmann S., Vandeleur C.L., Schmid M., Barth J., Eichholzer M. (2017). Loneliness is adversely associated with physical and mental health and lifestyle factors: Results from a Swiss national survey. PLoS ONE.

[47] Eisenberger N. (2012). The pain of social disconnection: Examing the shared neural underpinnings of physical and social pain. Nat. Rev. Neurosci..

[48] Matthews T., Danese A., Caspi A., Fisher H.L., Goldman-Mellor S., Kepa A., Moffitt T.E., Odgers C.L., Arseneault L. (2018). Lonely young adults in modern Britain: Findings from an epidemiological cohort study. Psychol. Med..

[49] Lambie-Mumford H., Dowler E. (2015). Hunger, food charity and social policy–challenges faced by the emerging evidence base. Soc. Policy Soc..

[50] Linares E. (2001). Food services for the homeless in Spain: Caritas programme for the homeless. Public Health Nutr..

